# Analysis of Residual Triflumizole, an Imidazole Fungicide, in Apples, Pears and Cucumbers Using High Performance Liquid Chromatography

**DOI:** 10.5487/TR.2008.24.1.087

**Published:** 2008-03-01

**Authors:** Sathya Khay, A. M. Abd El-Aty, Jeong-Heui Choi, Jae-Han Shim

**Affiliations:** 112 Natural Products Chemistry Laboratory, Institute of Agricultural Science and Technology, College of Agriculture and Life Science, Chonnam National University, 300 Yong-Bong dong, Buk-gu, Gwangju, 500-757 Korea; 212 Department of Veterinary Pharmacology and Toxicology, College of Veterinary Medicine, Konkuk University, Seoul, 143-701 Korea; 312 Department of Pharmacology, Faculty of Veterinary Medicine, Cairo University, 12211 Giza, Egypt

**Keywords:** Fungicide, Analysis, Liquid chromatography

## Abstract

The present study was conducted to monitor the level of triflumizole residues in fruits (apple and pear) and vegetable (cucumber) samples in order to assess risk posed by the presence of such residues to the consumer. Triflumizole was applied at a recommended dose rate to apple and pear pulps and to a cucumber sample. The samples were collected at harvesting time following several treatments (three and/or four treatments). Triflumizole was extracted with methanol and re-extracted into dichloromethane. The presence of triflumizole was determined by HPLC with UV detection at 238 nm following the cleanup of the extract by open preparative chromatographic column with Florisil. The versatility of this method was evidenced by its excellent linearity (> 0.999) in the concentration range between 0.2 and 4.0 mg/kg. The mean recoveries evaluated from the untreated samples spiked at two different fortification levels, 0.1 and 0.4 mg/kg, and ranged from 87.5 ± 0.0 to 93.3 ± 2.6 for the tested fruits and vegetable, respectively, and the repeatability (as relative standard deviation) from three repetitive determinations of recoveries were no larger than 6%. The calculated limit of detection was 0.02 mg/kg and the minimum detectable level of 4 ng for triflumizole was easily detected. When triflumizole was sprayed onto the apple trees three times at 50-40-30 and 40-30-21 days prior to harvesting and four times onto the pear trees at 40-30-21-14 days prior to harvesting, the mean residual amounts of 0.05 and 0.06 mg/kg for apples and pears, respectively, were not detected in all of the treatments. When the cucumber sample was fumigated four times at 7, 5, 3 and 1 day prior to harvesting, the mean residual amount was not detectable. Triflumizole can be used safely when sprayed (wettable powder, 30% active ingredient) and fumigated (10%) 4 times at 14 and 1 day prior to harvesting to protect the fruits and vegetable, respectively.

## INTRODUCTION

The role of agrochemicals in modern agricultural practices is continuously evolving, and their contribution to crop protection and environmental policy is becoming increasingly significant. The use of pesticides is essential in modern day agricultural practices; however, due to their biocidal activity and potential health risk to consumers, the control of the presence of pesticide residues in foods is a growing source of concern for the general population (Torres *et al.*, 1996). Triflumizole, (E)-4-chloro-α,α,α-trifluoro-N-(1-imidazol-1-yl-2-propoxy-ethyli dene)-o-toluidine, is an imidazole fungicide used to control scabs and rusts on fruit trees as well as the growth of powdery mildews on many crops (Nakata *et al.*, 1991; Pis'mennaia, 1991; Roberts and Hutson, 1999; Tomlin, 2000). The chemical structure of triflumizole is shown in Fig. 1. The monitoring of pesticide residues is crucial to the proper assessment of human

†These two authors contributed equally to this paper.

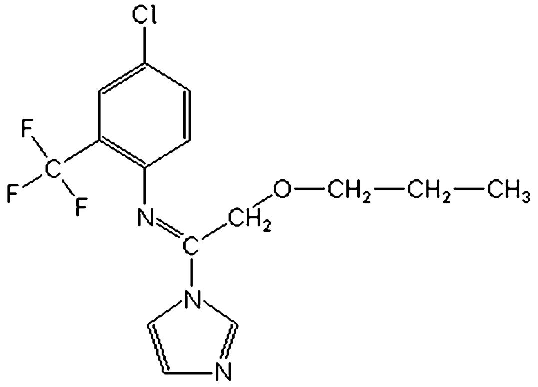

**Fig. 1.** Chemical structure of triflumizole.

exposure to pesticides through foods. The Pesticide Management Regulatory Agency (PMRA) of Health Canada (Canada Gazette, 2004) has determined the MRLs of triflumizole to be 0.5 ng/kg in apples, pears and cucumbers. These values are consistent with those established by the United States Environmental Protection Agency (CFR 40 part 180 Tolerances and Exemptions from Tolerances for Pesticide Chemical in Food, 2005).

In Korea, fruit production is primarily concentrated on five major fruits, including apples and asian pears. Fungal infections are serious diseases specific to apples and pears. Rainfall and high temperatures are among the factors that favor the spread of the disease. Although diseased fruit does not pose a risk to human health, it is necessary to protect apples and pears from such diseases.

Cucumbers, commonly used for pickling or slicing, have become one of the most popular and extensively grown crops worldwide. China leads in cucumber production, followed by India, Russia, the U.S., and various European countries. In addition, there is some greenhouse production of cucumbers, particularly in Korea, Japan and northern Europe, and to a lesser degree in the U.S. and some parts of the middle east. A wide spectrum of disease organisms (bacteria, fungi, viruses) can infect cucumbers and result in serious crop losses. Powdery mildew (*Erysiphe*) is a common fungal disease (Tomlin, 2000) affecting cucumbers grown particularly in greenhouse environments, where high temperatures and humidity constitute ideal conditions for the development of diseases. Based on frequent harvests, an effective pesticide program with recommended material must be followed to control the spread of disease and to ensure that the crop has no or low residues below the maximum residue levels established by various governments. This study reports on the determination of the residual level of the triflumizole pesticide in fruits and vegetable crops using an HPLC-UV detection system.

## MATERIALS AND METHODS

***Chemicals and reagents.*** Pure standard triflumizole (99.6%) was kindly provided by Kyong Nong Co. (Seoul, Korea). Methanol, acetonitrile, acetone and hexane were of pesticide residue analytical grade and were purchased from Merck (Darmstadt, Germany). Anhydrous sodium sulfate was supplied by Merck KGaA (Darmstadt, Germany). Dichloromethane was obtained from DC Chemical Co., Ltd (Seoul, Korea) and Celite 545 was obtained from Daejeon Chemicals and Materials Co., Ltd, Korea. All other chemicals and reagents were of analytical grade, unless otherwise noted.

***Pesticide application and sampling.*** Apple and pear samples were harvested in bulk from Chonnam University Farm, Korea. Triflumizole (wettable powder, WP; 30% active ingredient) sprays were applied to the apple and pear trees. The trees were planted 4.0 m apart from each other in rows, and the distance between the rows was 5.0 m. The approximate average height of the trees was 2.5 m, with an average trunk diameter of 5.5 cm. The orchard was managed throughout the duration of the experiment using the standard production practices outlined in the guidelines established by the Korean Ministry of Agriculture and Forestry and the College of Agriculture and Life Science, Chonnam National University. Triflumizole (10 g) diluted in 20 liters of water was applied to the test blocks with a tractor-mounted sprayer (Turbo Mist, Penticton, BC) equipped with a handgun. Each block consisted of three apple and three pear trees. A control block in the same orchard consisted of three trees of similar size and growth pattern. The treatments were administered according to the manufacturer's protocol as follows: spraying triflumizole to apple and pear trees at 50-40-30, 40-30-21 and at 40-30-21-14 days prior to harvesting. Cucumbers at the greenhouse of Chonnam National University were fumigated 4 times with 10-day intervals between doses by triflumizole tablet (10%). The temperature and humidity ranged from 24 to 28°C and 62 to 85%, respectively. The samples were harvested 1, 3, 5 and 7 days after the final fumigation. All of the fruit and vegetable samples (control and treated samples) were chopped after harvesting to obtain thoroughly mixed homogenates, packed with plastic bags, labeled and stored at -24°C until analysis. The samples were analyzed within one month after the harvest.

***HPLC analysis.*** The samples were analyzed using an HPLC system (Kontron, Italy) equipped with Detector 335A (Kontron, Italy). The column used was a Nova-Pak® $C_{18}$ 3.9 × 300 mm × 4 μm column (Waters, USA). The injection volume was set at 20 μl. The detection wavelength was 238 nm. The mobile phase was acetonitrile/deionized water/methanol (6/3/1, v/v/v) and the flow rate was 1ml/min. The retention time of triflumizole was 7.7 min under these conditions.

***Sample extraction.*** Twenty grams of each apple, pear and cucumber sample was weighed in a 250 ml homogenizer cup and extracted with 100 ml of methanol. The mixture was macerated at 7000 rpm for 5 min in a high speed homogenizer (WiseMix™ HG15A, Daihan Scientific, Seoul, Korea). The homogenate was then filtered through filter paper (Whatman® 90 mm Diameters, No. 6, England) under suction and celite 545 (Daejeon Chemicals and Materials Co., Daejeon, Korea) on a porcelain Büchner funnel. The filtrate was then quantitatively transferred into a 500 ml separatory funnel. Fifty milliliters of dichloromethane were added to 100 ml of a 10% sodium chloride solution, which was shaken vigorously for 3 min and then allowed to separate into two phases. The bottom layer, organic phase, was collected into a round-bottomed flask and the water layer was re-extracted with additional dichloromethane (50 ml). The dichloromethane extract was evaporated to dryness on a rotary evaporator (Büchi Rotavapor R-114, Germany) in a water bath of 40°C (Büchi Waterbath B-480, Germany).

***Cleanup.*** A slurry of 10 g of Florisil in *n*-hexane was poured into an open preparative chromatographic column (18 mm diameter, 65 cm length, 200 ml volume, Paul Marienfeld GmbH & Co. KG, Lauda-Königshofen, Germany), which was connected to a conical flask as a reservoir. Five grams of anhydrous sodium sulphate were added to the top of the adsorbent. The column was washed with 50 ml of *n*-hexane and the elute fraction was discarded. The dried sample residue was dissolved in 5 ml of acetone/*n*-hexane (5 : 95, v/v), and the flask was then washed with another 5 ml of acetone/*n*-hexane and placed on the top of the column. A 50 ml aliquot of a mixture of acetone/*n*-hexane (5/95, v/v) was added to wash the sample, followed by 150 ml of acetone/*n*-hexane (15/85, v/v) to elute the pesticide from the Florisil column. The eluate was passed through anhydrous sodium sulphate, evaporated at 40°C and dissolved in acetonitrile to obtain a final volume up to 2 ml.

***Quantitative analysis.*** The triflumizole standard (10 mg) was weighed and dissolved in 100 ml of acetonitrile to make a 100 mg/kg stock solution. The stock solutions were diluted with the appropriate amounts of acetonitrile to obtain concentrations corresponding to 0.2, 0.5, 1, 2 and 5 mg/kg of triflumizole residues and these were analyzed by HPLC. The correlation coefficient was above 0.999. The residue concentrations were calculated by the calibration curve generated from the peak areas obtained from the results of the standard injection.

***Recovery test.*** Recovery studies were conducted with the apple, pear and cucumber samples. The pesticide solution (2.0 ml) was spiked to homogeneous samples (20 g) at the concentration of 0.1 or 0.4 mg/kg. The resulting samples were homogenized for 5 min and then submitted to extraction, cleanup, and HPLC-UV analysis as previously described. Each recovery test was repeated three times. The blank and spiked samples were analyzed routinely in each analytical series to ensure the reliability of the method.

## RESULTS AND DISCUSSION

Reliable analytical methods are important aspects in the monitoring of pesticide residue levels to ensure human and environmental safety. Residue monitoring methods utilizing high-performance liquid chromatography (HPLC) with ultraviolet (UV) detection have been previously used to monitor the levels of triflumizole in crops and vegetables (Shiga *et al.*, 1987; Cai *et al.*, 1999). The methods generally include an extraction of the analytes with suitable organic solvents. The initial purification of the extracts is accomplished using organic partitioning. Further cleanup is achieved using a Florisil column (Shiga *et al.*, 1987). The analyte was then separated and determined using HPLC with UV detection at 238 nm. This report briefly summarizes the method validation data for the various sample matrices and the method for determining triflumizole levels by HPLC-UV. It also describes modifications of the previous method that were necessary to obtain satisfactory results for some of the additional matrices.

The following modifications were made in the present study. Whatman paper No 6 and celite 545 were used for the extraction to improve the filtration of the fine particles, which was not mentioned in the previous method (Shiga *et al.*, 1987). The reduced pressure in a water-bath at less than 40°C was replaced by a rotary vacuum evaporator worked at 40°C for dryness. The final eluent used in the cleanup was a 150 ml mixture of acetone/*n*-hexane (15 : 85, v/v) that replaced the 100 ml of acetone/*n*-hexane (10 : 90, v/v) previously used. These modifications were made to allow for adequate cleanup and increased recoveries. The HPLC conditions include the Nova-Pak $C_{18}$ column and mobile phase consisting of acetonitrile/deionized water/methanol (6 : 3 : 1, v/v/v) that replaced the reversed phase column Nucleosil $C_{18}$ and the eluent acetonitrile-carbonate buffer. The Nova-Pak column used under these conditions gave a reproducible retention time (7.7 min) in comparison to Nucleosil (12 min).

The chromatograms corresponding to triflumizole in the spiked apple, pear and cucumber samples are shown in Fig. 2. There were no interfering peaks observed near the peaks of triflumizole, allowing for the quantitation of the analyte. The retention time of triflumizole was 7.7 min.

The percent recoveries generated during the validation of the analytical methods are shown in Table 1. The analysis revealed that the average recoveries of triflumizole residues from apple, pear and cucumber samples using the HPLC analytical method were 87.5% ± 0.0 to 91.7% ± 5.0, 87.6% ± 5.2 to 93.3% ± 2.8 and 84.2% ± 1.5 to 90.5% ± 4.6 for 0.1 and 0.4 mg/kg, respectively. Our findings are consistent with the previously reported validation data (Shiga *et al.*, 1987).

The agricultural products were analyzed with the proposed method. The pesticide involved was clearly detected without any serious interference. The detected pesticide was identified by comparing its authentic retention time and UV spectra to the reference standards. The limit of detection (LOD) was 0.02 mg/kg for triflumizole. No detectable residues were found in the pear and cucumber samples. The residue level of the positive apple samples was lower than 0.1 mg/kg (Table 2) and most of the residues were below the maximum residue limits (MRLs). In Korea and Japan, the maximum residue limits of triflumizole in apples, pears and cucumbers are 2.0, 2.0 and 1.0 mg/kg, respectively. The triflumizole residue levels found in our study were much lower than those of the MRLs established by Korea Food and Drug Administration and Japan.

In conclusion, the experiment on the determination of triflumizole residues in fruits and vegetables using high-

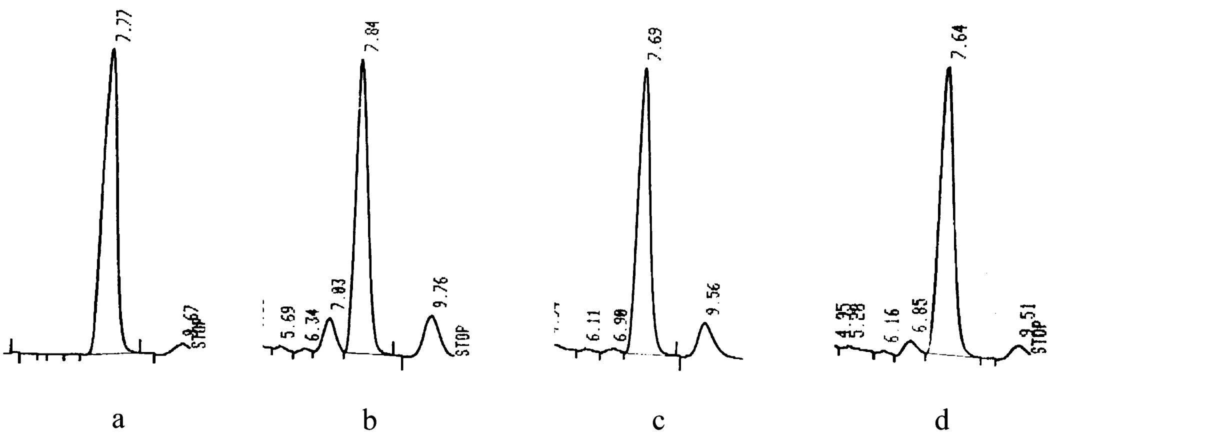

**Fig. 2.** Chromatogram of the triflumizole standard in acetonitrile (a), apple (b), pear (c), and cucumber (d) samples spiked with 4-mg/kg.

**Table 1.** Recoveries and detection limit of triflumizole using HPLC

| Sample | Spiked level (mg/kg) | Recovery (%) | | | | Detection limit (mg/kg) | Minimum detectable level (ng) |
|---|---|---|---|---|---|---|---|
| | | 1 | 2 | 3 | Mean ± RSD | | |
| Apple | 0.1 | 87.5 | 87.5 | 87.5 | 87.5 ± 0.0 | 0.02 | 4.0 |
| | 0.4 | 89.0 | 89.0 | 97.0 | 91.7 ± 5.0 | | |
| Pear | 0.1 | 82.8 | 91.8 | 88.2 | 87.6 ± 5.2 | | |
| | 0.4 | 96.3 | 91.8 | 91.8 | 93.3 ± 2.8 | | |
| Cucumber | 0.1 | 92.9 | 85.7 | 92.9 | 90.5 ± 4.6 | | |
| | 0.4 | 82.9 | 84.5 | 85.3 | 84.2 ± 1.5 | | |

Triplicates for each concentration.
RSD: Relative standard deviation.

**Table 2.** Residual triflumizole in apple, pear and cucumber samples

| Spray frequency | Interval before harvest | Interval from last spray | Residue (mg/kg) | | | | MRL* (mg/kg) |
|---|---|---|---|---|---|---|---|
| | | | A | B | C | Average residue | |
| Apple | | | | | | | |
| Control | - | - | ND | ND | ND | ND | |
| 3 | 50-40-30 | 30 | ND | ND | ND | ND | 0.5 |
| 3 | 40-30-21 | 21 | 0.02 | 0.05 | 0.08 | 0.05 ± 0.024 | |
| 4 | 40-30-21-14 | 14 | 0.07 | 0.06 | 0.04 | 0.05 ± 0.015 | |
| Pear | | | | | | | |
| Control | - | ND | ND | ND | ND | ND | |
| 3 | 50-40-30 | ND | ND | ND | ND | ND | 0.5 |
| 3 | 40-30-21 | ND | ND | ND | ND | ND | |
| 4 | 40-30-21-14 | ND | ND | ND | ND | ND | |
| Cucumber | | | | | | | |
| Control | - | - | ND | ND | ND | ND | |
| 4-time spraying with 10-day interval | 31-21-11-1 | 1 | ND | ND | ND | ND | 0.5 |
| | | 3 | ND | ND | ND | ND | |
| | | 5 | ND | ND | ND | ND | |

ND: not detected.
*MRL referred to the Korean Food and Drug Administration.

performance liquid chromatography was successfully accomplished and disclosed that all triflumizole applications to apples, pears and cucumbers had no detectable levels of the residues. Therefore, we could recommend these treatment schedules for triflumizole use in apples, pears and cucumbers and suggest that these products are not harmful to consumers. However, the triflumizole residue analysis in other varieties of fruits and vegetables needs further evaluation.

## ACKNOWLEDGEMENT

This work was supported by the BK21 program of the Ministry of Education and Human Resources through the Department of Agricultural Chemistry at Chonnam National University, Republic of Korea.
